# Semantic diversity is best measured with unscaled vectors: Reply to Cevoli, Watkins and Rastle (2020)

**DOI:** 10.3758/s13428-021-01693-4

**Published:** 2021-09-29

**Authors:** Paul Hoffman, Matthew A. Lambon Ralph, Timothy T. Rogers

**Affiliations:** 1 School of Philosophy, Psychology & Language Sciences, University of Edinburgh, 7 George Square, Edinburgh, EH8 9JZ UK; 2 MRC Cognition and Brain Sciences Unit, University of Cambridge, Cambridge, UK; 3 University of Wisconsin-Madison, Madison, WI USA

**Keywords:** Semantic diversity, Lexical ambiguity, Polysemy

## Abstract

Semantic diversity refers to the degree of semantic variability in the contexts in which a particular word is used. We have previously proposed a method for measuring semantic diversity based on latent semantic analysis (LSA). In a recent paper, Cevoli et al. (2020) attempted to replicate our method and obtained different semantic diversity values. They suggested that this discrepancy occurred because they scaled their LSA vectors by their singular values, while we did not. Using their new results, they argued that semantic diversity is not related to ambiguity in word meaning, as we originally proposed. In this reply, we demonstrate that the use of unscaled vectors provides better fits to human semantic judgements than scaled ones. Thus we argue that our original semantic diversity measure should be preferred over the Cevoli et al. version. We replicate Cevoli et al.’s analysis using the original semantic diversity measure and find (a) our original measure is a better predictor of word recognition latencies than the Cevoli et al. equivalent and (b) that, unlike Cevoli et al.’s measure, our semantic diversity is reliably associated with a measure of polysemy based on dictionary definitions. We conclude that the Hoffman et al. semantic diversity measure is better-suited to capturing the contextual variability among words and that words appearing in a more diverse set of contexts have more variable semantic representations. However, we found that homonyms did not have higher semantic diversity values than non-homonyms, suggesting that the measure does not capture this special case of ambiguity.

**Supplementary Information:**

The online version contains supplementary material available at 10.3758/s13428-021-01693-4.

## Introduction

A wealth of work indicates that the contexts in which we encounter words have powerful effects on how we learn and represent their meanings (Firth, 1957; Landauer & Dumais, 1997; Schwanenflugel et al., 1988). We and other researchers use the term semantic diversity to refer to the degree of semantic variability in the contexts in which a particular word is used (Hoffman et al., 2011; Jones et al., 2012). In a paper published in BRM in 2013, we proposed a measure of semantic diversity based on latent semantic analysis (LSA)(Hoffman et al., 2013). We argued that words that appear in a wide range of semantically diverse contexts are likely to have high variability in their meanings, aligning semantic diversity with notions of lexical ambiguity and, in particular, polysemy. We proposed our semantic diversity measure provided a sensitive and objective measure of the degree to which a word’s meaning is ambiguous.

In a recent paper, Cevoli et al., (2020) presented data that challenge this interpretation of semantic diversity. They attempted to replicate our derivation of semantic diversity values by applying LSA to the British National Corpus (BNC). However, their results diverged somewhat from those we had reported: they found a correlation of *r* = 0.72 between their SemD values and those we originally reported.^1^ Cevoli et al. suggested that differences stemmed from a specific difference in the methods used when applying LSA: after performing singular value decomposition (SVD) to obtain vector representations of each context, they scaled these vectors by their singular values while we did not. Cevoli et al. opted to use scaled vectors in their computations “because scaling by the singular values is a key feature of LSA methods” (p. 4). Using their scaled semantic diversity values, they assessed the relationship between lexical ambiguity and semantic diversity in stimuli used in published studies that manipulated polysemy and homonymy. They found that their scaled semantic diversity values did not co-vary with either polysemy or homonymy. They concluded that semantic diversity is not related to ambiguity in a word’s meaning and is instead determined by “a word’s spread across topics and types of contexts”.

In this reply, we argue that the central question here—how components of a term-document matrix as extracted by SVD relate to important psycholinguistic constructs—is an empirical one rather than an analytic one. We demonstrate that, when LSA is applied to the BNC, unscaled vectors are better predictors of human semantic judgements than scaled vectors and better capture the semantic relationships between different contexts in the corpus. We then replicate Cevoli et al.’s analyses of the effects of semantic diversity on word recognition performance and in stimulus sets used to investigate ambiguity, comparing their semantic diversity measure with ours. We refer to the semantic diversity values reported in our 2013 paper as H13_SemD and the new values computed by Cevoli et al. as C20_SemD. We show that H13_SemD has similar effects on word recognition as C20_SemD but greater predictive power. In three stimulus sets used to investigate lexical ambiguity, we show that greater polysemy is reliably associated with higher H13_SemD values, but is unrelated to C20_SemD. Homonymy, on the other hand, is unrelated to either semantic diversity measure. Together these analyses suggest that unscaled vectors like those described by Hoffman et al. (2013) provide a more useful estimate of the psychological constructs of interest. We end by considering implications for what semantic diversity is and how it should be measured.

## The effect of vector scaling on latent semantic vectors

The method Cevoli et al. used to produce the C20_SemD values differed from our procedures in two ways. First, Cevoli et al. lemmatised the tokens in the BNC prior to analysis and excluded function words; in contrast, we used the original inflected wordforms in the corpus, including function words. Second, they scaled the vectors representing each context in the corpus by the singular values. It is worth noting that when Cevoli et al. followed our procedures exactly (inflected corpus and no scaling) they obtained semantic diversity values that were extremely highly correlated with the H13_SemD values (*r* = 0.98). Thus, we can be confident that any differences between C20_SemD and H13_SemD values stem from either the lemmatisation or the vector scaling. Cevoli et al. showed that lemmatisation had little effect on semantic diversity values: they calculated semantic diversity values from the lemmatised corpus (but without vector scaling) and these correlated with H13_SemD values at *r* = 0.93. The discrepancy comes therefore from the scaling.

To understand the significance of this difference, it is necessary to briefly outline the steps involved in LSA. LSA is performed on a corpus partitioned into discrete documents or contexts. To investigate semantic diversity, we and Cevoli et al. used the BNC partitioned into contexts of 1000 words. The corpus is used to generate a term-by-context matrix which logs the number of occurrences of each term in each context. Values in the matrix are weighted (here using a log-entropy scheme) then decomposed into *k* orthogonal components using SVD, which re-represents the original matrix as a product of three matrices. For *n* words and *m* contexts, and extracting *k* orthogonal components, the procedure approximates the original data A as:

$$ A\sim =U\kern0.15em S\kern0.15em V $$

where U is an *n* x *k* matrix that expresses each term as a *k*-dimensional vector, S is a diagonal matrix containing singular values for each of the *k* dimensions , and V is a *k* x *m* matrix that expresses each context as a *k*-dimensional vector. Typically *k* is small relative to both *n* and *m*, so the SVD represents a lower-rank approximation of the original data. The current work uses *k* = 300, so each word and context is represented in a 300-dimensional space.

In LSA, the effect of these steps is to represent terms and contexts in a high-dimensional “semantic space” in which terms that are used in similar contexts have similar vectors and, similarly, contexts that contain similar terms have similar vectors. The cosine similarity between two terms or two contexts (in their respective spaces) captures information about their semantic relatedness, following the general principle that meaning similarity can be inferred from patterns of lexical co-occurrence in language (Firth, 1957; Landauer & Dumais, 1997; Lund & Burgess, 1996; Mikolov et al., 2013). To estimate the semantic diversity of a word, we took all of the contexts in which that word appeared and computed the mean pairwise cosine similarity between them. High mean similarity between contexts indicates that the word is used in related, semantically homogeneous contexts that discuss similar topics, while a low level of similarity indicates greater diversity in usage.

In some natural-language workflows, the vectors are scaled by their singular values before cosines are computed. This simply means that the first element of each vector is multiplied by the first singular value, the element by the second singular value and so on, and the cosines are then computed between the scaled vectors rather than the original vectors. As a consequence, dimensions that have high singular values “count more” in the estimation of pairwise similarity between vectors. Scaling is described as a standard part of the analysis workflow in the *Handbook of LSA*(Martin & Berry, 2007). In psycholinguistic research, however, such a step amounts to a hypothesis about how the latent spaces estimated by SVD relate to the psychological similarities encoded in the human semantic system. Specifically, scaling assumes that dimensions are strongly important for reconstructing the original data matrix (captured in diagonal matrix S above) are likewise more important in determining psycho-semantic similarity between two terms or two contexts. An alternative hypothesis is that psycho-semantic similarities are better expressed by the unscaled embeddings. Neither hypothesis is transparently true analytically, so we see this as an empirical question.

In the Python library Cevoli et al. used to perform LSA (scikit-learn) scaling is performed automatically, but this step is by no means universal. For example, the commonly used LSA Boulder website gives users the option of comparing terms without singular value scaling (“term space”) or with scaling (“document space”) (Dennis, 2007). Likewise the landmark *Psychological Review* paper in which Landauer and Dumais (1997) first proposed LSA as a tool for understanding human semantic representation appears not to have used scaling.^2^ In general, scaling is thought to be advantageous when evaluating similarity between a term vector and a context vector, for example when using keywords to search for documents (Hu et al., 2007; Martin & Berry, 2007). Psycholinguistic applications of LSA, however, rarely require such comparisons of term vectors with context vectors. Typically, LSA is used to compare one term vector with another, in order to estimate the semantic relatedness of the terms (e.g., Pereira et al., 2016). Likewise when computing semantic diversity, we compare context vectors with other context vectors but not with term vectors. In these situations, we argue that it is best to assess empirically whether scaling improves the quality of the semantic representations.

To this end, we performed two empirical investigations comparing scaled and unscaled vectors. Although there are many ways in which vectors could potentially be scaled (e.g., by the square root of the singular values or by their inverse), here we focused on the simple case of multiplication by singular values, as used by Cevoli et al. We began by assessing the ability of scaled vs. unscaled term vectors to predict human judgements of semantic relatedness for word pairs. Following Pereira et al. (2016), we obtained three sets of human semantic relatedness ratings: the MEN dataset (Bruni et al., 2014), the WordSim-353 dataset (Finkelstein et al., 2001) and the SimLex-999 dataset (Hill et al., 2015). We computed Spearman’s rank correlations between the rated similarities of word pairs and the cosine similarities between the word’s vectors, computed for the following sets of vectors:

1. BNC_unscaled: term vectors from LSA applied to the BNC exactly as described in Hoffman et al., *without* vector scaling
2. BNC_scaled: Term vectors from LSA applied to the BNC exactly as described in Hoffman et al., but *with* vector scaling
3. Wiki_unscaled and Wiki_scaled. Term vectors from LSA applied to a corpus of Wikipedia articles,^3^ with and without vector scaling. We included these to test whether the effect of scaling is consistent across corpora.
4. Word2vec: These were publicly available vectors (https://code.google.com/p/word2vec/), obtained by training the word2vec neural network (Mikolov et al., 2013) with the 10-billion word Google News dataset. Pereira et al. (2016) found that this set of vectors was the best predictor of human semantic judgements in a comparison of “off-the-shelf” vector spaces. We included these as a benchmark indicator of the current gold-standard for distributional semantic models.

The correlations between human ratings and vector cosines are shown in Table 1. As expected, word2vec vectors provided the closest fit to the human data. More importantly, unscaled vectors were more strongly correlated with human ratings than scaled vectors, for both the BNC vectors and those derived from the Wikipedia corpus. To further test whether scaled or unscaled BNC vectors provide a better fit to human data, we computed partial Spearman’s correlations between the human ratings and the BNC_unscaled cosines, while controlling for shared variance with BNC_scaled cosines. In each case, a strong correlation remained (MEN: ρ = 0.42, *p* < 0.001; WordSim-353: ρ = 0.37, *p* < 0.001; LexSim-999: ρ = 0.21, *p* < 0.001). However, there were no significant correlations between human ratings and BNC_scaled cosines, when controlling for BNC_unscaled cosines (MEN: ρ = 0.02, *p* = 0.24; WordSim-353: ρ = 0.09, *p* = 0.10; SimLex-999: ρ = – 0.06, *p* = 0.06). These data clearly indicate that unscaled BNC vectors are better predictors of human judgements of semantic relatedness than scaled vectors. Similar effects in the Wikipedia vectors suggest that this may be a general result.

**Table 1:** Spearman correlations between human semantic relatedness judgements and vector cosines

	MEN	WordSim-353	SimLex-999
BNC_unscaled	0.72	0.64	0.27
BNC_scaled	0.65	0.57	0.19
wiki_unscaled	0.69	0.67	0.26
wiki_scaled	0.65	0.60	0.21
word2vec	0.78	0.69	0.44

**Fig. 1:**
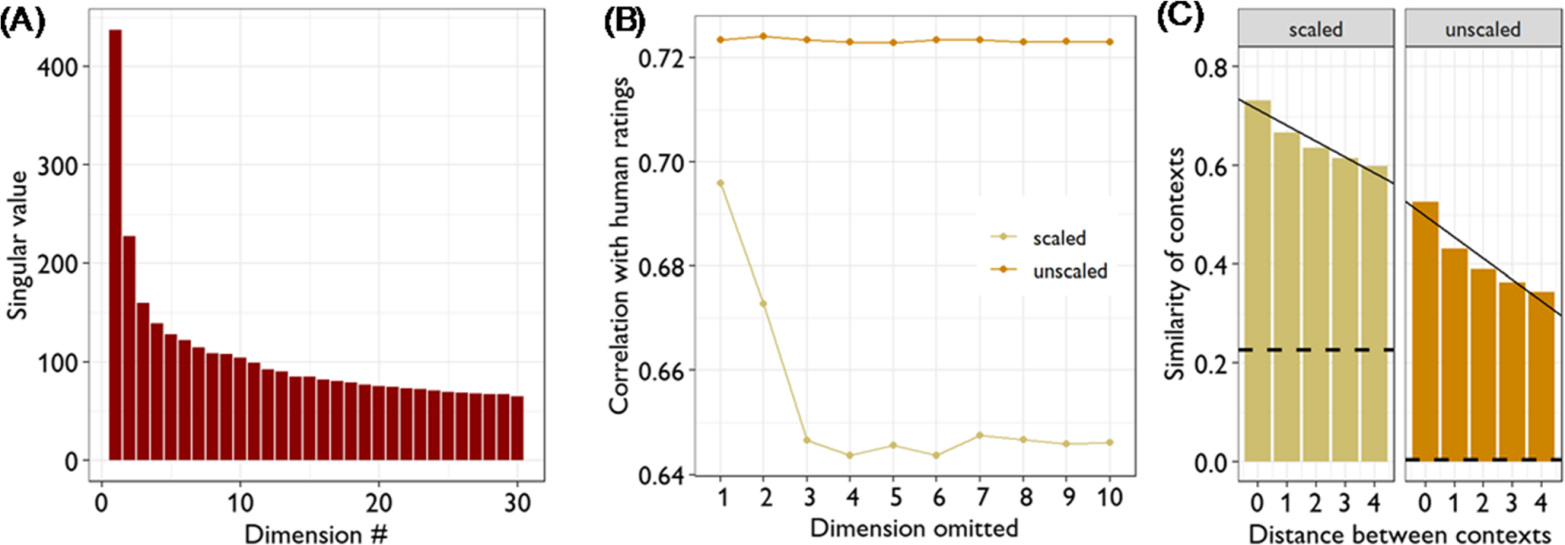
Comparisons of scaled and unscaled LSA vectors. **a** Singular values for the first 30 dimensions when singular value decomposition is applied to BNC data. **b** Correlations between MEN semantic relatedness ratings and cosine similarity when different dimensions were omitted from the LSA vectors. Omitting dimensions had no effect on unscaled vectors, but omitting either of the first two dimensions considerably improved the fit to human data for scaled vectors. **c** Mean cosine similarity between neighbouring contexts, as a function of their distance in the corpus. Both sets of vectors show reduced similarity as distance increases, but this effect is significantly stronger for the unscaled vectors. The *dashed lines* indicate the mean similarity between two contexts chosen at random from the corpus (calculated by permuting the order of contexts in the corpus and calculating the mean similarity between adjacent contexts in this permuted corpus)

Why do unscaled vectors perform better than scaled ones? Figure 1a shows the singular values for the first 30 dimensions in the LSA space derived from the BNC. The first two dimensions have much larger singular values than those of later dimensions. Scaling therefore gives these dimensions considerably more weight when computing cosines between vectors and our investigations indicate that this is detrimental. We repeated our calculations of the correlations between MEN ratings and BNC vectors but this time we systematically omitted one of the 300 dimensions each time. The results when omitting dimensions one to ten are shown in Fig. 1b. We found that if we omitted either of the first two dimensions, the performance of the scaled vectors improved while the unscaled vectors were unaffected. In fact, if we excluded *both* of the first two dimensions, the scaled and unscaled vectors gave near-indistinguishable correlations with relatedness judgements (0.724 vs. 0.721). These results strongly suggest that scaled vectors are inferior because they give too much weight to the information contained in the first two dimensions of the latent semantic space. We do not know why these two dimensions have much higher weights than the others, but we have observed that values on the first dimension are strongly correlated with lexical frequency (|*r*| = 0.81; this was not true for any of the later dimensions). Thus it appears that the first dimension largely encodes information about how often terms appear in the corpus. Since term frequency information is unlikely to be helpful in determining semantic relationships, over-weighting this dimension is detrimental to performance.

So far, we have established that vector scaling is not desirable when considering term vectors that represent the meanings of individual words. However, the calculation of semantic diversity is based on context vectors, not term vectors. We could not perform the same analyses for context vectors because there are no data for human judgements of similarity between contexts in the BNC. Instead, we assessed the context vectors by investigating how the semantic similarity between two contexts is predicted by their distance from one another in text. It seems intuitively reasonable to assume that passages of text that appear close to one another in a document are likely to discuss similar topics while those that are more distant will tend to be less related. Foltz et al. (1998) demonstrated this phenomenon empirically by using LSA to analyse semantic similarity between different paragraphs in textbooks. They found that adjacent paragraphs in a text tended to have high cosine similarity values and that similarity between paragraphs decreased as the distance between paragraphs in the text increased. They argued that these effects were indicative of the gradual shifts in topic that occur in coherent texts. Here, we assessed whether scaled or unscaled vectors best capture similar effects in the BNC.

The BNC consists of a little over 3000 distinct documents obtained from different sources. When we performed LSA on the corpus, we sub-divided these documents into smaller contexts of 1000 words in length. This means that adjacent contexts almost always came from adjoining portions of the same document. We would therefore expect these to be closely related in meaning. As distance between contexts increases, we would expect their similarity to decrease, because they feature text drawn from increasingly distant portions of each document (and also because it becomes more likely that the two contexts have been sourced from different documents).

The results of this analysis are shown in Fig. 1c. For each context, we measured the cosine similarity with its near neighbours, separated by a distance of 0 contexts (adjacent) up to 4 contexts in the corpus. As expected, similarity decreased with increasing distance between contexts. However, this rate of decrease was significantly steeper for the unscaled vectors, suggesting that these vectors better capture the expected relationship between proximity and meaning. This was supported by the finding of a significant interaction between vector type and distance in a linear mixed effect model predicting cosine values (*B* = 0.0079, *SE* = 0.00011, *t*(788964) = 72.8, *p* < 0.001; the model included a random intercept for contexts).

Figure 1c shows another advantage of unscaled vectors. The dashed lines show the mean cosine similarity for two contexts selected at random from the corpus. For the unscaled vectors the mean is close to zero, confirming the intuitive expectation that two randomly chosen contexts should have no semantic relationship between them. However, the value for scaled vectors is substantially higher, indicating that scaled vectors show similarity between randomly paired contexts not expected to have any semantic relationship. Interestingly, we found that this undesirable effect was again due to the over-weighting of the first two dimensions in the scaled vectors. If we omitted the first two dimensions from the scaled vectors, the mean similarity for randomly selected contexts fell to zero.

When using context vectors to compute SemD values, one potential advantage of scaling could be to increase the stability of SemD values over the number of dimensions used in the calculation. Applications of LSA typically use the first 300 dimensions of the semantic space (following the seminal work of Landauer & Dumais, 1997) but this is an arbitrary choice and other values could have been chosen. It could be argued that scaling makes SemD values less sensitive to the choice of number of dimensions, since the influence of adding later dimensions is minimised by their low singular values. To test the stability of unscaled SemD values, we compared SemD values computed using unscaled vectors while varying the number of dimensions (50, 100, 150, 200, 250, and 300). SemD values were highly stable over changes in dimensionality: pairwise comparisons revealed very high correlations in every case (*r >* 0.94). SemD values computed from scaled vectors were also highly stable (*r* > 0.97). Thus scaling is not necessary to achieve SemD values that are insensitive to vector length.

To summarise, in this first section we have argued that scaling of LSA vectors is not essential when computing semantic diversity and that the decision whether to perform this step should be made empirically. Our empirical investigations indicated (a) that unscaled term vectors provide better fits to human semantic relatedness judgements, (b) that unscaled context vectors better capture the expected pattern of decreasing semantic similarity with increasing distance between contexts, and (c) that only unscaled vectors satisfy the expectation that two contexts selected at random from the corpus will share no semantic relationship on average. Our analyses indicated that scaled vectors are inferior because the scaling process gives too much influence to the first two dimensions in the latent semantic space. Thus, we conclude the H13_SemD values, computed from unscaled vectors, are likely to provide more reliable estimates of semantic diversity than the C20_SemD values recently published by Cevoli et al. Having established this, we proceeded to replicate Cevoli et al.’s key analyses using H13_SemD values rather than C20_SemD data.

## Effects of semantic diversity on word recognition

**Fig. 2:**
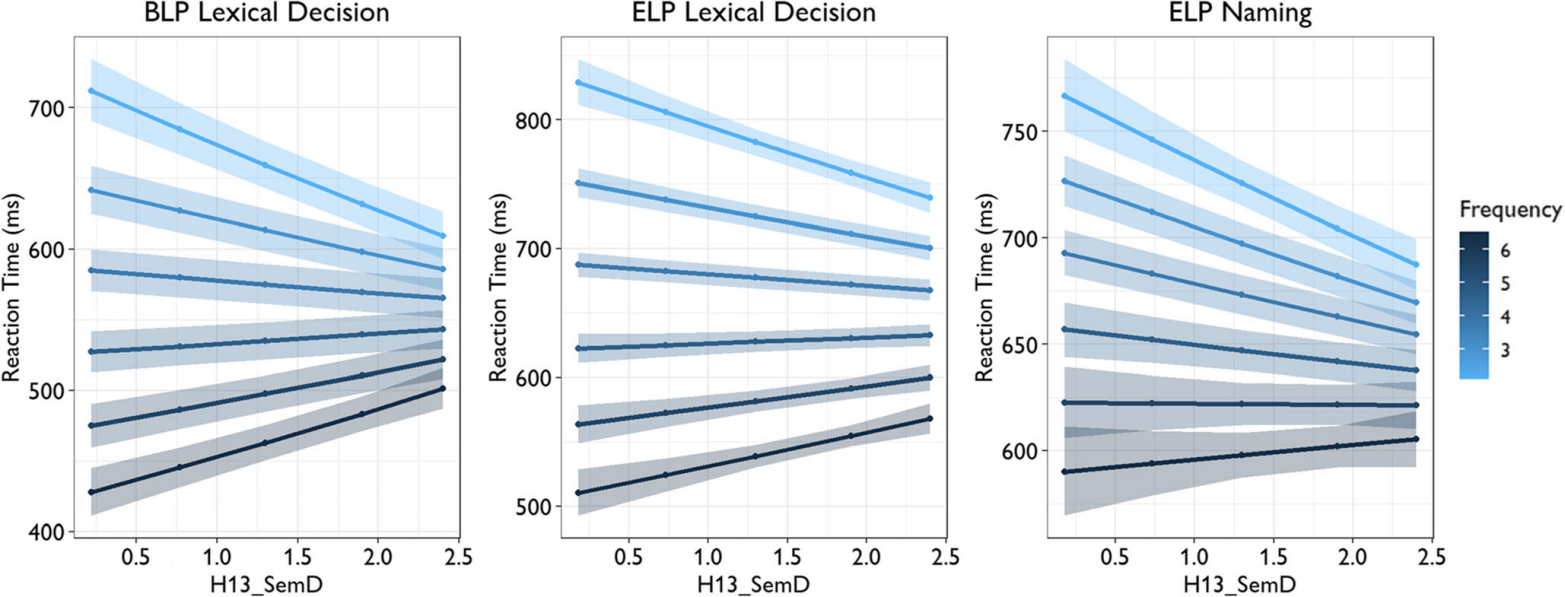
Effects of H13_SemD on word recognition latencies at various levels of word frequency. Word frequency values indicate log counts in the BNC

Cevoli et al. investigated how C20_SemD, as well as its interaction with word frequency, age of acquisition, and word length, predict word recognition performance in two large open datasets: the British Lexicon Project (Keuleers et al., 2012) and the English Lexicon Project (Balota et al., 2007). Here we tested whether similar results would be obtained for H13_SemD values, focusing on reaction time data. We fitted linear mixed effects models predicting lexical decision and word naming latencies from semantic diversity and other psycholinguistic variables, using the code provided by Cevoli et al. on their OSF page (https://osf.io/7hxvu/). Analyses were restricted to words that had both C20_SemD and H13_SemD values, to ensure that models were always fitted on the same data. The effects of H13_SemD at different levels of word frequency are shown in Fig. 2(for full results of each model, see Supplementary Table 1). Overall, words with higher H13_SemD values were recognised more quickly, as found in previous studies (Hoffman & Woollams, 2015; Hsiao et al., 2020; Hsiao & Nation, 2018). There was, however, a significant interaction with frequency in every dataset. The facilitatory effect of H13_SemD was largest for low-frequency words and was absent or possibly even reversed for the highest frequency words. These results are very similar to those reported by Cevoli et al. using C20_SemD data.

We also compared how well models that used C20_SemD vs. H13_SemD were able to predict reaction times. We compared AIC and BIC values for models that included each semantic diversity measure, and its interaction with other variables, with a baseline model that included neither. As shown in Table 2, the inclusion of H13_SemD provided greater improvements over the baseline than the inclusion of C20_SemD (indicated by lower AIC and BIC values). These results suggest that the H13_SemD has greater explanatory power than C20_SemD when considering word recognition latencies.

**Table 2:** Model fit statistics for models predicting word recognition latencies with and without semantic diversity

Dataset:	BLP: Lexical decision		ELP: Lexical decision		ELP: Word naming	
Model	AIC	BIC	AIC	BIC	AIC	BIC
Baseline	– 116838	– 116690	– 12888	– 12735	– 170609	– 170469
Baseline + C20_SemD and its interactions	– 116903	– 116713	– 12980	– 12784	– 170671	– 170488
Baseline + H13_SemD and its interactions	**– 116960**	**– 116770**	**– 12992**	**– 12795**	**– 170778**	**– 170596**

The lowest AIC/BIC values in each case (indicating best model fit) are underlined

## Relationship between semantic diversity and lexical ambiguity

In this section, we replicated Cevoli et al.’s critical analyses of the relationships between semantic diversity and word ambiguity, using our H13_SemD values in place of the C20_SemD data. Cevoli et al. investigated three stimulus sets used by Rodd et al. (2002) and Armstrong and Plaut (2016) to disentangle effects of homonymy and polysemy on word recognition.

**Fig. 3:**
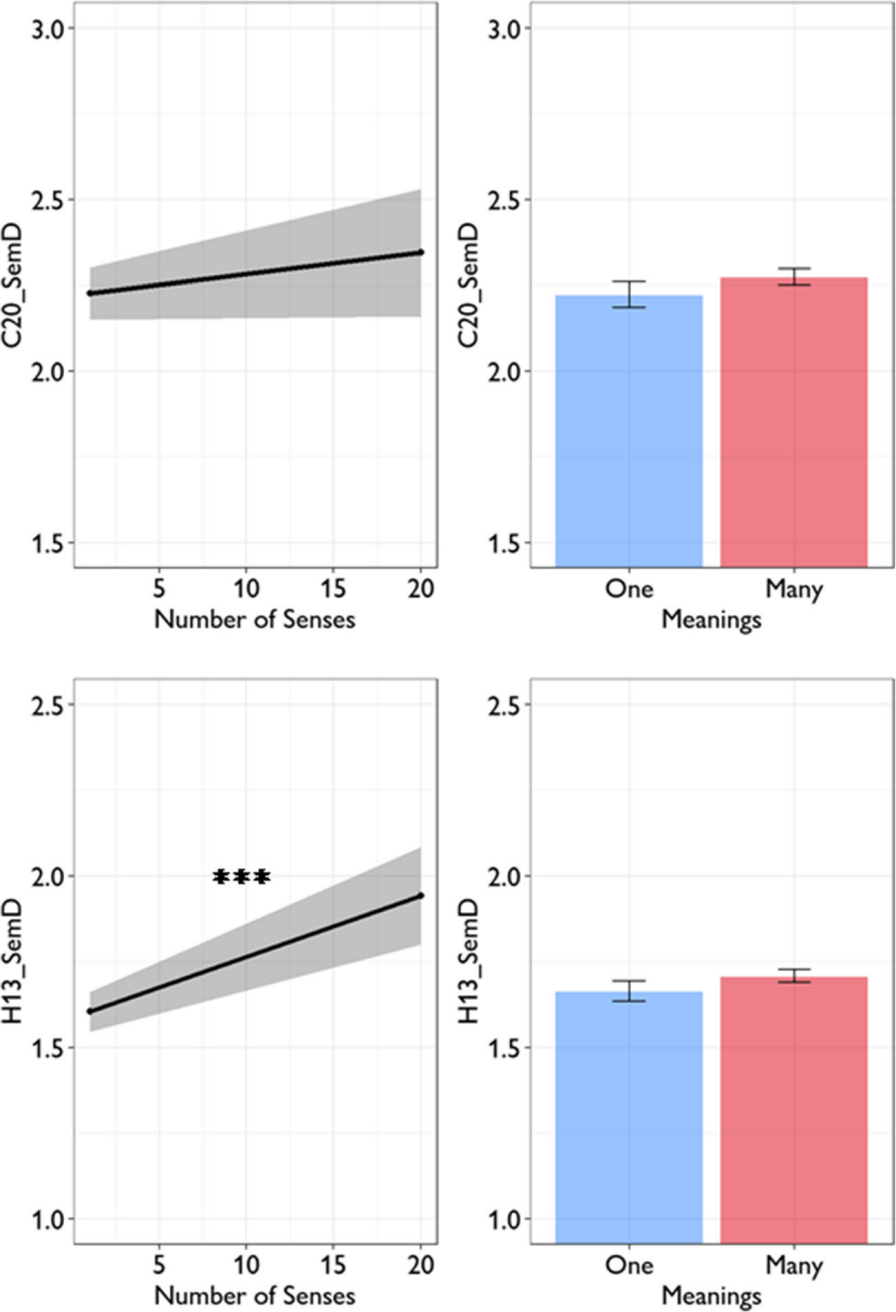
Effects of lexical ambiguity on semantic diversity in stimuli used in Rodd et al.’s Experiment 1

Experiment 1 of Rodd et al. (2002) used 184 words that varied in ambiguity. Rodd et al. used entries in the Wordsmyth online dictionary to quantify ambiguity. This dictionary distinguishes between the number of etymologically unrelated meanings a word has (providing a measure of homonymy) and the number of related senses it has (providing a measure of polysemy). To investigate how semantic diversity was related to these two types of ambiguity, Cevoli et al. fitted a linear regression model with C20_SemD as the dependent variable and number of meanings (binarised: one or many) and number of senses as predictors. A number of other psycholinguistic properties were also included as predictors. In Table 3, we show results of their model alongside the same analysis using H13_SemD as the dependent variable. Cevoli et al. found that neither number of meanings nor number of senses predicted C20_SemD values. However, when H13_SemD data was used instead, there was a significant effect of number of senses: words with more senses tended to have higher H13_SemD values (see Fig. 3). The H13_SemD model therefore replicates the result originally reported by Hoffman et al. (2013): words that are highly polysemous, as measured by the number of senses they have in the Wordsmyth dictionary, have higher SemD values, even after controlling for other relevant psycholinguistic properties. There was no effect of number of meanings.

**Table 3:** Linear regression models predicting semantic diversity in stimuli used in Rodd et al.’s Experiment 1

Dependent variable:	C20_SemD		H13_SemD	
Predictor	*β*	*t*	*β*	*t*
Number of meanings	0.086	1.06	0.079	1.21
Number of senses	0.081	0.95	0.244	3.51***
Orthographic neighbours (Coltheart’s N)	0.031	0.34	0.058	0.78
Frequency	0.262	3.34**	0.280	4.39***
Length	0.113	1.22	– 0.025	0.33
Concreteness	– 0.248	3.41***	– 0.405	6.93***

C20_SemD model: *df* = 163, *R*^*2*^ = 0.21. H13_SemD model: *df* = 164, *R*^*2*^ = 0.48. * = *p* < 0.05; ** = *p* < 0.01; *** = *p* < 0.001

**Fig. 4:**
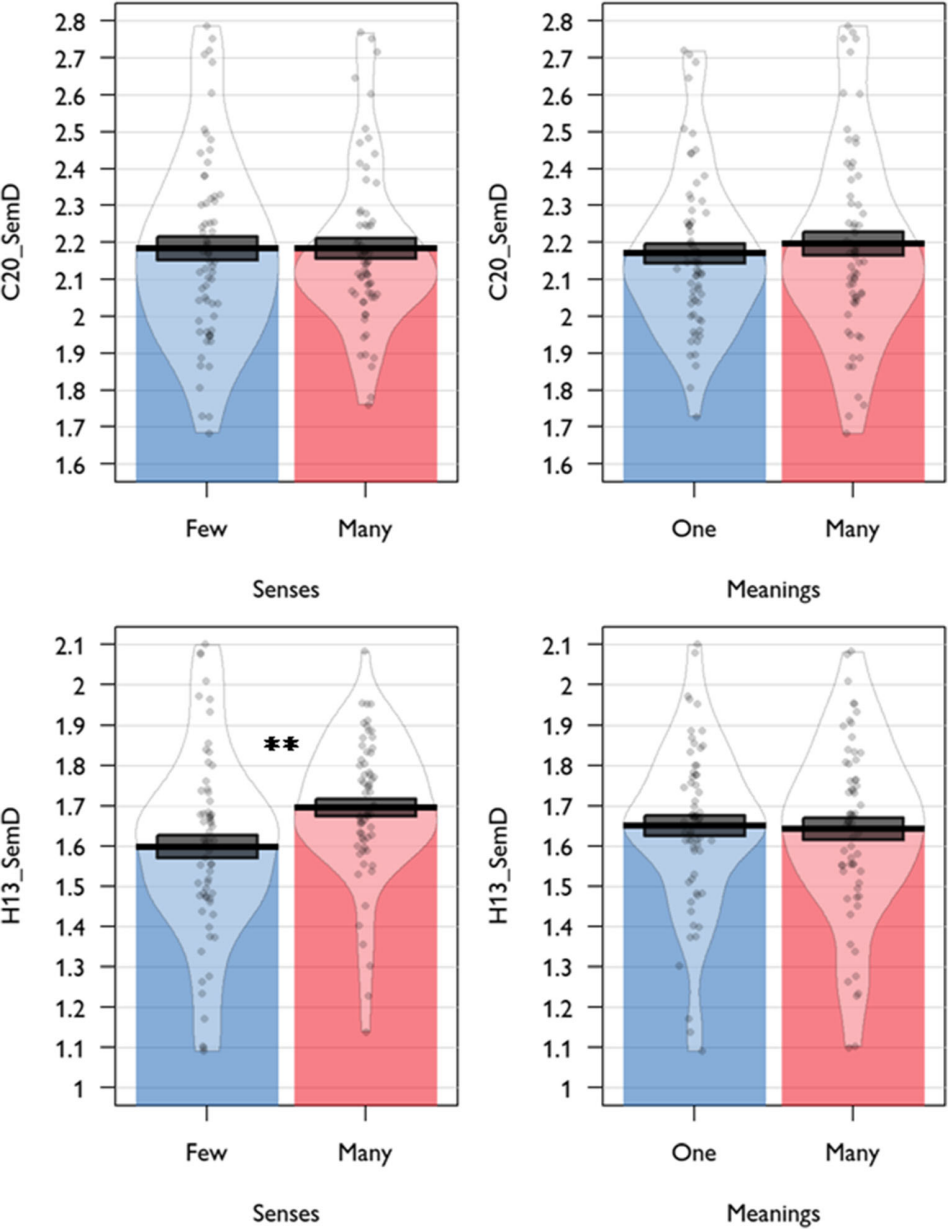
Effects of lexical ambiguity on semantic diversity in stimuli used in Rodd et al.’s Experiment 2

Rodd et al.’s (2002) Experiment 2 employed a 2 x 2 factorial manipulation of homonymy and polysemy, again based on Wordsmyth definitions. Mean C20_SemD and H13_SemD values for words in each condition are shown in Fig. 4. Cevoli et al. investigated how C20_SemD varied across conditions using an ANCOVA model that included meanings and senses conditions as between-words factors, while covarying for word frequency and length. As shown in Table 4, this model revealed no effects of ambiguity on C20_SemD; however, polysemy had a significant effect on H13_SemD values. As expected, words in the Many Senses condition had higher H13_SemD values than those in the Few Senses condition. These models do not control for concreteness, which tends to be highly predictive of semantic diversity (see Table 3). When we added concreteness as a covariate to the ANCOVA model, the effect of number of senses on H13_SemD remained highly significant (*F*(1,121) = 8.77, *p* = 0.004). There was no effect of number of meanings on H13_SemD values, similar to the Experiment 1 stimuli.

**Table 4:** Results of ANCOVA predicting semantic diversity in stimuli used in Rodd et al.’s Experiment 2

Dependent variable:	C20_SemD		H13_SemD	
Effect	*F*	*p*	*F*	*p*
Number of meanings	0.000	0.54	0.052	0.82
Number of senses	0.385	0.99	7.293	0.008**
Meanings * Senses	0.018	0.18	0.334	0.56
Frequency	1.835	0.10	6.144	0.015*
Length	2.813	0.89	0.012	0.91

C20_SemD model: *df* = 1,122. H13_SemD model: *df* = 1,122. * = *p* < 0.05; ** = *p* < 0.01; *** = *p* < 0.001

**Fig. 5:**
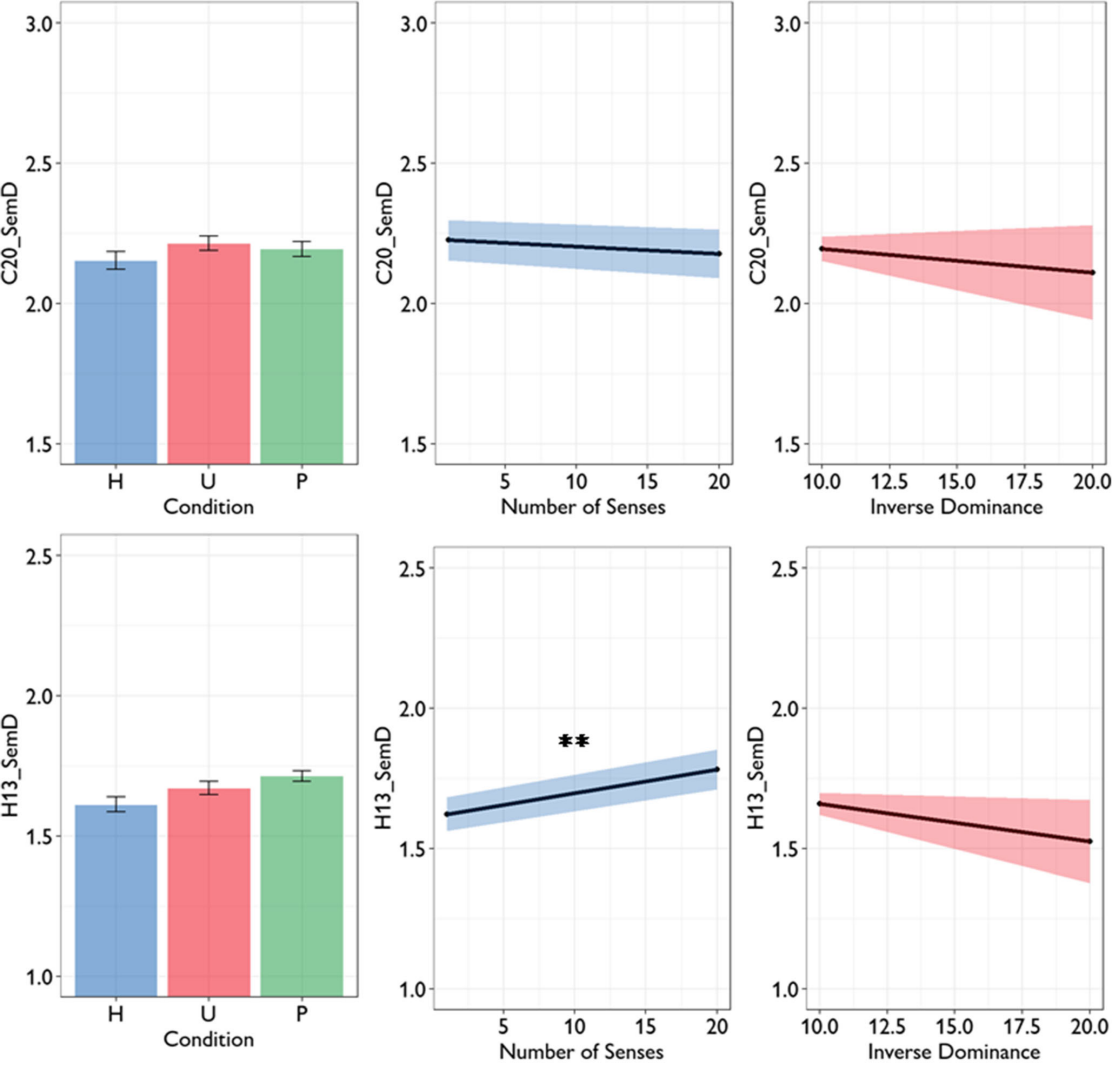
Effects of lexical ambiguity on semantic diversity in stimuli used by Armstrong and Plaut Left panel shows mean semantic diversity values for words classified as Homonyms (H), Unambiguous (U) and Polysemous (P)

The final stimulus set was used by Armstrong and Plaut (2016) to investigate homonymy and polysemy effects. Cevoli et al. performed two linear regression analyses investigating semantic diversity effects in the Armstrong and Plaut (2016) stimuli. In the first, they adopted a binary classification of polysemy (few vs. many senses). When we replicated this analysis, we found that this binary classification predicted neither C20_SemD (*β =*– 0.038, *p =* 0.57) nor H13_SemD (*β =* 0.119, *p =* 0.11; for full model results, see Supplementary Table 2). But what if we used a continuous measure of polysemy, following the approach taken to analyse Rodd et al.’s (2002) Experiment 1? When we replaced the binary polysemy variable with the number of senses in Wordsmyth, we found that this was a significant positive predictor of H13_SemD (*β =* 0.158, *p =* 0.034), though not of C20_SemD (*β =* -0.052, *p =* 0.45; for full model results, see Supplementary Table 3). Furthermore, concreteness was not included as a covariate in Cevoli et al.’s analyses, despite having large effects on semantic diversity. When we included concreteness, the effect of number of senses on H13_SemD was highly significant, while no effect was observed on C20_SemD (see Table 5). These effects are plotted in Fig. 5. Thus, the degree to which a word is polysemous once again predicted its H13_SemD value, once other relevant variables were taken into account. We note that concreteness was a much better predictor of H13_SemD than C20_SemD and it’s possible that greater sensitivity to this semantic variable partly explains why H13_SemD values are more related to measures of polysemy (since highly polysemous words tend to be more abstract).

**Table 5:** Linear regression models predicting semantic diversity in polysemy stimuli used by Armstrong & Plaut

Dependent variable:	C20_SemD		H13_SemD	
Predictor	*β*	*t*	*β*	*t*
Number of senses	– 0.044	0.65	0.179	2.62**
Frequency	0.522	6.38***	0.138	1.64
Orthographic neighbours (OLD20)	– 0.012	0.13	– 0.115	1.17
Number of syllables	0.041	0.52	0.006	0.07
Length	– 0.069	0.68	0.133	1.32
Familiarity (residual)	– 0.425	4.86***	0.030	0.33
Concreteness	– 0.130	1.84	– 0.423	5.85***

C20_SemD model: *df* = 179, *R*^*2*^ = 0.24. H13_SemD model: *df* = 178, *R*^*2*^ = 0.22. * = *p* < 0.05; ** = *p* < 0.01; *** = *p* < 0.001

In their final analysis, Cevoli et al. investigated whether homonymy predicted C20_SemD values. Rather than classify words as either unambiguous or homonyms, here they followed Armstrong and Plaut (2016) in adopting a continuous measure of homonym polarity. They used a measure they called inverse dominance, in which unambiguous words take the minimum value of 10, fully balanced homonyms (where both meanings are equally frequent in the language) take the maximum value of 20 and polarised homonyms fall between these two extremes. Inverse dominance was entered into a model predicting C20_SemD along with other psycholinguistic variables. When we replicated these analyses, we found that inverse dominance did not predict either semantic diversity variable (see Supplementary Table 4), with the addition of concreteness producing similar results (see Table 6 and Fig. 5). These results converge with the previous analyses in showing that homonyms do not reliably differ from unambiguous words in their H13_SemD values.

**Table 6:** Linear regression models predicting semantic diversity in homonymy stimuli used by Armstrong & Plaut

Dependent variable:	C20_SemD		H13_SemD	
Predictor	*β*	*t*	*β*	*t*
Inverse dominance	– 0.060	0.88	– 0.113	1.60
Frequency	0.490	6.11***	0.149	1.76
Orthographic neighbours (OLD20)	– 0.005	0.05	– 0.019	0.19
Number of syllables	– 0.010	0.12	0.023	0.27
Length	– 0.090	0.88	– 0.044	0.41
Familiarity (residual)	– 0.257	2.89**	0.080	0.86
Concreteness	– 0.170	2.30*	– 0.385	4.96***

C20_SemD model: *df* = 173, *R*^*2*^ = 0.25. H13_SemD model: *df* = 173, *R*^*2*^ = 0.19. * = *p* < 0.05; ** = *p* < 0.01; *** = *p* < 0.001

## Discussion

In a recent paper, Cevoli et al. (2020) disputed the method we used to estimate semantic diversity in Hoffman et al. (2013) and provided a different interpretation of this variable based on their own semantic diversity measure. Here, we have presented new data that challenge their conclusions. First, we found that scaling the LSA vectors by their singular values has a negative effect on the quality of the resulting semantic representations. On this basis, we argue that C20_SemD values, derived from scaled vectors, provide a less valid measure of semantic diversity than our original H13_SemD values. Second, we replicated Cevoli et al.’s analyses of semantic diversity effects in word recognition, substituting our H13_SemD values for their C20_SemD. Like them, we found a positive effect of semantic diversity on word recognition performance, modulated by frequency. However, H13_SemD was a better predictor of word recognition than C20_SemD. Finally, and most importantly, we replicated Cevoli et al.’s analyses of the relationship between semantic diversity and lexical ambiguity. Cevoli et al. found that ambiguity did not predict C20_SemD and argued that semantic diversity is unrelated to variation in a word’s meaning. H13_SemD, conversely, reliably covaried with polysemy in every stimulus set, though it was not related to homonymy. We conclude that, when measured appropriately, semantic diversity is closely related to the level of polysemous variability in a word’s semantic representation.

Based on the results reported here, we recommend that researchers continue to use H13_SemD as a measure of semantic diversity. But what is semantic diversity? At the most basic level, we believe it provides a quantitative measure of the degree to which the different contexts in which a word is used are semantically distinct from one another. To this extent, we believe we are largely in agreement with Cevoli et al., who say:

“Overall, our analyses lead us to suggest that the metric defined by Hoffman et al. (2013) is a measure of a word’s spread across topics and types of contexts, rather than a measure of the diversity of a word’s contextual meaning. This metric is insensitive to the diversity of a word’s meanings; instead, it captures general information about the range of reading situations in which a word might be encountered. Words that are high in Hoffman et al.’s (2013) semantic diversity metric are well distributed across topics and types of contexts, while words that are low in this semantic diversity metric are specific to particular contexts.”

Where we diverge from Cevoli et al. is in claiming that semantic diversity *also* provides useful information about the level of contextual variability in a word’s meaning, in addition to variation in the contexts themselves. Here we use “meaning” to refer to the semantic information that is activated upon processing of a word. Due to their contextual promiscuity, we propose that high semantic diversity words are associated with a wide range of possible semantic states. This explains why there is a reliable relationship between the number of senses a word has in the Wordsmyth dictionary and its H13_SemD value: the list of senses captures in some discrete fashion the breadth of possible semantic states a word may take. In typical language settings, the context preceding a word will constrain the semantic state it engages, in line with models that view construal of meaning at the sentence level as an incremental process (McClelland et al., 1989; Rabovsky et al., 2018). However, when words are presented in isolation, as they often are in psycholinguistic studies, this constraint is not available and we assume that words of high diversity consequently activate noisy and under-specified semantic representations (for a computational simulation of this idea, see Hoffman & Woollams, 2015). This explanation accounts for the observed negative effects of semantic diversity in tasks that require deeper semantic processing than mere recognition (Hoffman & Woollams, 2015; Hsiao et al., 2020; Johns et al., 2016; Mak et al., 2021).

For recognition tasks such as lexical decision, words with high semantic diversity enjoy a processing advantage (Hoffman & Woollams, 2015; Hsiao et al., 2020; Jones et al., 2012). Our theory accounts for this by positing that, although the semantic activation elicited by these words is less precise, it is generated more rapidly and that this aids recognition (Hoffman & Woollams, 2015; for similar arguments applied to polysemous words, see Armstrong & Plaut, 2008; Rodd et al., 2004). Cevoli et al. propose a different “textual” account for these effects, suggesting that low diversity words may be harder to process because their appearance in a restricted range of contexts means that they are unfamiliar to some people. For example, one will rarely, if ever, encounter the low diversity word *crampon* unless one is exposed to specialised discourse or text on the topic of mountaineering. Thus, most participants will be slow to recognise this word due to limited exposure. This account neatly explains why the semantic diversity effect is larger for low-frequency words, since higher-frequency words are more likely to be encountered by everyone from time to time, despite their lack of contextual breadth. It also predicts greater individual variability in recognition of low-frequency words of low semantic diversity, a prediction which could be tested in future work. We find this account plausible; it seems likely that exposure effects of this kind contribute to word recognition, in addition to the semantically driven effects described above. However, it is not clear how Cevoli et al.’s “textual” view would account for *poorer* processing of highly diverse words in semantic tasks, and we do not see this as a complete account of how semantic diversity affects processing.

Semantic diversity appears to have a different relationship with different forms of ambiguity. We found that H13_SemD was reliably associated with polysemy, as indexed by a dictionary-derived measure of the number of related senses a word has. However, homonyms with two or more *unrelated* meanings did not have elevated H13_SemD values compared with single-meaning words. Thus, the measure does not appear to be sensitive to the particular, and less common, form of ambiguity present in homonyms. In fact, Hoffman et al. (2013) anticipated this. We noted that the contextual uses of homonyms may cluster into two distinct sets of contexts relating to the two different meanings (e.g., for *bark*, a dog-related set and a tree-related set). While we would expect the similarity of contexts in different clusters to be low, similarities within each cluster may be very high. This means that the overall semantic diversity value for a homonym will depend, amongst other factors, on the separation of the two clusters in semantic space, on the degree of contextual variation within each cluster (i.e., how polysemous each meaning is), and on the relative sizes of the clusters (i.e., when one meaning is much more frequent than the other, the majority of the pairwise comparisons will reflect variation within the dominant meaning). Thus, even if we accept the assumption that the different meanings of homonyms have distinct contextual environments, this form of ambiguity is not straightforwardly captured by the semantic diversity measure.

Cevoli et al. go further and argue that semantic diversity is not sensitive to homonymy because their different meanings are not reliably associated with distinct context vectors. To test this hypothesis, they took three homonyms (*calf, mole,* and *pupil*), extracted half of their appearances in the BNC and manually classified each appearance according to which of their meanings was implied. For each word, they then investigated how well the contexts associated with each meaning formed coherent clusters, measuring clustering with a Calinski-Harabasz score that represents the ratio between within-cluster dispersion and between-cluster dispersion. The scores for *calf, mole,* and *pupil* were 3.28, 2.08, and 4.43, indicating that between-cluster dispersion was between two and four times greater than within-cluster dispersion in these test cases. Cevoli et al. describe these scores as “relatively low” but they are between two and four times higher than control analyses where they assigned the meaning labels to contexts randomly (0.92, 1.13, and 0.97, respectively). How high should they be in order to infer that context vectors cluster based on the homonym’s meaning? We don’t have a definitive answer to this, but we find these results rather encouraging, especially considering that they used scaled vectors which, as we have demonstrated, are less well suited to capturing semantic relationships in the BNC than unscaled vectors. We also note that the lowest level of clustering was observed for *mole,* but the classification for this word combined two rather different interpretations (animal and spy) into a single meaning, which may have hampered identification of coherent clusters. Thus, we reject the idea that LSA is inherently insensitive to contextual differences between different word meanings. Future work could investigate how often the different meanings of homonyms occupy semantically distinct contextual environments and the circumstances in which LSA context vectors best capture these differences.

To conclude, our analyses indicate that the original H13_SemD measure, without scaling of context vectors, are a more appropriate way of measuring semantic diversity than Cevoli et al.’s alternative. We found that H13_SemD has a positive effect on word recognition performance, particularly for low-frequency words and, critically, that words with more senses tend to have higher H13_SemD values. Our interpretation of the latter result is that words that appear a more diverse set of linguistic contexts become associated with more variable semantic representations. This variability may be either beneficial or detrimental to lexical processing, depending on the task involved and the degree of precision required from the semantic system.

## Supplementary Information

ESM 1 (PDF 81 kb)

## Footnotes

1 We are able to replicate this correlation using the data uploaded to OSF by Cevoli et al.

2 No mention of scaling is made in their Method and the following comment suggests that it was not performed: “It is worth noting that the cosine measure sums the degree of overlap on each of the dimensions of representation of the two entities being compared. In LSA, *the elements of this summation have been assigned equal fixed weights,* but it would be a short step to allow differential weights for different dimensions in dynamic comparison operations, with instantaneous weights influenced by, for example, attentional, motivational, or contextual factors.” (p. 218; emphasis added).

3 This corpus was obtained and processed as follows. A 2018 dump of the entire English Wikipedia site was downloaded here: https://linguatools.org/tools/corpora/wikipedia-monolingual-corpora/. XML tags and disambiguation articles were removed. Then 70% of the articles were selected at random and submitted to LSA using the Text to Matrix Generator (Zeimpekis & Gallopoulos, 2006), with each article treated as a separate context. Log-entropy weighting was applied prior to singular value decomposition and 300 dimensions were retained.
