# Supplementary material for: Semantic diversity is best measured with unscaled vectors: Reply to Cevoli, Watkins and Rastle (2020)
Source: Behav Res Methods. 2021 Sep 29;54(4):1688–700. doi: 10.3758/s13428-021-01693-4 (PMC9374602; doi:10.3758/s13428-021-01693-4)
Supplement: Supplementary file 1 — (PDF 81 kb) [file 13428_2021_1693_MOESM1_ESM.pdf]

Supplementary Table 1: Linear mixed effects models predicting word recognition latencies using each semantic diversity measure

Model:		C20_SemD			H13_SemD		
Dataset	Fixed effects	*B*	s.e.	*t*	*B*	s.e.	t
BLP Lexical Decision	SemD	<0.01	<0.01	-1.64	<0.01	<0.01	-6.00***
	Freq	-0.05	<0.01	-48.58***	-0.05	<0.01	-50.56***
	Length	<0.01	<0.01	9.09***	<0.01	<0.01	9.22***
	AoA	0.04	<0.01	45.86***	0.04	<0.01	47.37***
	SemD*Freq	<0.01	<0.01	7.35***	<0.01	<0.01	9.02***
	SemD*Length	<0.01	<0.01	-2.83**	<0.01	<0.01	-0.94
	SemD*AoA	<0.01	<0.01	3.37***	<0.01	<0.01	3.45***
	AoA*Freq	-0.02	<0.01	-18.13***	-0.01	<0.01	-15.93***
	AoA*Length	<0.01	<0.01	-0.29	<0.01	<0.01	-0.62
	Length*Freq	<0.01	<0.01	1.54	<0.01	<0.01	1.08
ELP Lexical Decision	SemD	<0.01	<0.01	-6.73***	<0.01	<0.01	-5.91***
	Freq	-0.05	<0.01	-52.19***	-0.05	<0.01	-56.42***
	Length	0.05	<0.01	67.88***	0.05	<0.01	68.06***
	AoA	0.05	<0.01	60.97***	0.05	<0.01	60.12***
	SemD*Freq	<0.01	<0.01	3.90***	<0.01	<0.01	6.12***
	SemD*Length	<0.01	<0.01	3.77***	<0.01	<0.01	4.28***
	SemD*AoA	<0.01	<0.01	-1.88	<0.01	<0.01	-0.59
	AoA*Freq	-0.01	<0.01	-15.09***	-0.01	<0.01	-14.10***
	AoA*Length	<0.01	<0.01	11.98***	0.01	<0.01	13.26***
	Length*Freq	<0.01	<0.01	-0.56	<0.01	<0.01	0.13
ELP Naming	SemD	<0.01	<0.01	-7.42***	<0.01	<0.01	-11.38***
	Freq	-0.03	<0.01	-27.25***	-0.03	<0.01	-28.91***
	Length	0.05	<0.01	61.96***	0.05	<0.01	62.43***
	AoA	0.05	<0.01	57.56***	0.05	<0.01	56.04***
	SemD*Freq	<0.01	<0.01	2.59**	<0.01	<0.01	3.74***
	SemD*Length	<0.01	<0.01	-1.09	<0.01	<0.01	1.88
	SemD*AoA	<0.01	<0.01	1.73	<0.01	<0.01	-0.13
	AoA*Freq	-0.01	<0.01	-16.34***	-0.01	<0.01	-15.24***
	AoA*Length	0.01	<0.01	16.74***	0.01	<0.01	17.33***
	Length*Freq	<0.01	<0.01	0.38	<0.01	<0.01	-0.11

** = $p < 0.05$; ** = $p < 0.01$; *** = $p < 0.001$.*

Supplementary Table 2: Linear regression models predicting semantic diversity in stimuli used by Armstrong & Plaut, using a binary measure of polysemy

Dependent variable:	C20_SemD		H13_SemD	
Predictor	*β*	*t*	*β*	*t*
Unambiguous vs. Polysemous	-0.038	0.57	0.119	1.61
Frequency	0.539	6.61***	0.247	2.74**
Orthographic neighbours (OLD20)	0.009	0.09	-0.057	0.53
Number of syllables	0.061	0.79	0.050	0.59
Length	-0.075	0.75	0.119	1.08
Familiarity (residual)	-0.467	5.51***	-0.128	1.37

*C20_SemD model: df = 180, R^2 = 0.23. H13_SemD model: df = 179, R^2 = 0.06. * = $p < 0.05$; ** = $p < 0.01$; *** = $p < 0.001$.*

Supplementary Table 3: Linear regression models predicting semantic diversity in stimuli used by Armstrong & Plaut, using a continuous measure of polysemy

Dependent variable:	C20_SemD		H13_SemD	
Predictor	*β*	*t*	*β*	*t*
Number of senses	-0.052	0.76	0.158	2.14*
Frequency	0.545	6.66***	0.229	2.54*
Orthographic neighbours (OLD20)	0.007	0.07	-0.052	0.49
Number of syllables	0.057	0.74	0.061	0.71
Length	-0.074	0.74	0.116	1.06
Familiarity (residual)	-0.469	5.53***	-0.121	1.30

*C20_SemD model: df = 180, R^2 = 0.22. H13_SemD model: df = 179, R^2 = 0.07. * = $p < 0.05$; ** = $p < 0.01$; *** = $p < 0.001$.*

Supplementary Table 4: Linear regression models predicting semantic diversity in stimuli used by Armstrong & Plaut, using inverse dominance

Dependent variable:	C20_SemD		H13_SemD	
Predictor	*β*	*t*	*β*	*t*
Inverse dominance	-0.063	0.91	-0.107	1.42
Frequency	0.530	6.69***	0.251	2.87**
Orthographic neighbours (OLD20)	0.014	0.14	0.031	0.29
Number of syllables	0.011	0.13	0.074	0.81
Length	-0.110	1.06	-0.086	0.75
Familiarity (residual)	-0.340	4.12***	-0.110	1.22

*C20_SemD model: df = 173, R^2 = 0.22. H13_SemD model: df = 174, R^2 = 0.07. * = $p < 0.05$; ** = $p < 0.01$; *** = $p < 0.001$.*
